# The Use of an External Cutting Guide for Patient-Specific Bone Grafting in Reverse Total Shoulder Arthroplasty: A Novel Technique

**DOI:** 10.1177/24715492231219566

**Published:** 2023-12-11

**Authors:** Graeme T Harding, Aaron J Bois, Joseph T Cavanagh, Martin J Bouliane

**Affiliations:** 1 Department of Orthopaedic Surgery, 3158 University of Alberta, Edmonton, AB, Canada; 2 Section of Orthopaedic Surgery, Department of Surgery, 70401 Cumming School of Medicine, University of Calgary, Calgary, AB, Canada; 3 McCaig Institute for Bone and Joint Health, University of Calgary, Calgary, AB, Canada

**Keywords:** glenoid deformity, reverse shoulder arthroplasty, bone graft, external cutting guide

## Abstract

Glenoid bone loss remains a substantial challenge in reverse shoulder arthroplasty and failure to address such bone loss may lead to implant malpositioning, instability and/or premature baseplate loosening. Currently, management of glenoid bone loss can be achieved by metal augmentation or bone grafting (ie, autograft or allograft). At the present time, options for creating and shaping glenoid bone grafts include free-hand techniques and simple reusable cutting guides that create the graft at a standard shape/angle. To our knowledge, there is no external guide available that enables surgeons to accurately prepare the bone graft to the desired dimensions/shape (ie, trapezoid or biplanar) to correct the glenoid deformity. In this article, we present a novel surgical technique that utilizes an external guide for creating a patient-specific bone graft to address glenoid deformity in the setting of reverse total shoulder arthroplasty.

## Introduction

Managing patients with substantial glenoid bone loss secondary to arthritis, cuff tear arthropathy (CTA) or dysplasia remains a challenge in reverse total shoulder arthroplasty (RSA).^1–3^ Large glenoid defects pose a risk for improper positioning and fixation of the glenoid baseplate and increase the risk of inferior scapular notching and perimeter impingement, premature glenoid component loosening and/or failure, and joint instability.^4,5^

Multiple techniques have been previously described for managing glenoid bone loss in the setting of RSA. In common practice, however, there are two primary options for managing large glenoid bone defects with little consensus on the optimal method. These options include metal augmentation of the glenoid baseplate (ie, standard wedge or patient-matched implant) or bone grafting of the glenoid defect. Preoperative planning software such as Blueprint™ (Stryker, Bloomington, MN) permits precise calculation of the angle, shape, and morphology of the required patient-specific bone graft (ie, “digital graft”). However, at the present time, there is no reproducible surgical technique available to create a patient-specific graft intraoperatively. Therefore, surgeons are dependent on using “free-hand” techniques to create a patient-specific graft, a method that is considered imprecise, technically demanding, and time-consuming.

To date, no optimal technique and instrumentation has been developed for the creation of this digitally generated patient-specific bone graft. The purpose of this study was to present a surgical technique, along with novel instrumentation, for the creation of a customized (ie, patient-specific) glenoid bone graft to address glenoid deformity in the setting of RSA.

## Surgical Technique

### Patient Evaluation

The patient is assessed in the clinic setting and a standard history is obtained, and a physical examination is performed taking note specifically of active and passive range of motion, deltoid and rotator cuff function, and noting the presence of any lag signs. Plain radiographs consisting of true anteroposterior and axillary views of the shoulder are obtained. Preoperatively, informed consent was obtained verbally from two patients for publication of the case details and accompanying imaging (ie, one patient case was used to outline each step of the surgical technique and a second patient case was used in the clinical results section).

### Preoperative Planning

**Figure 1.**
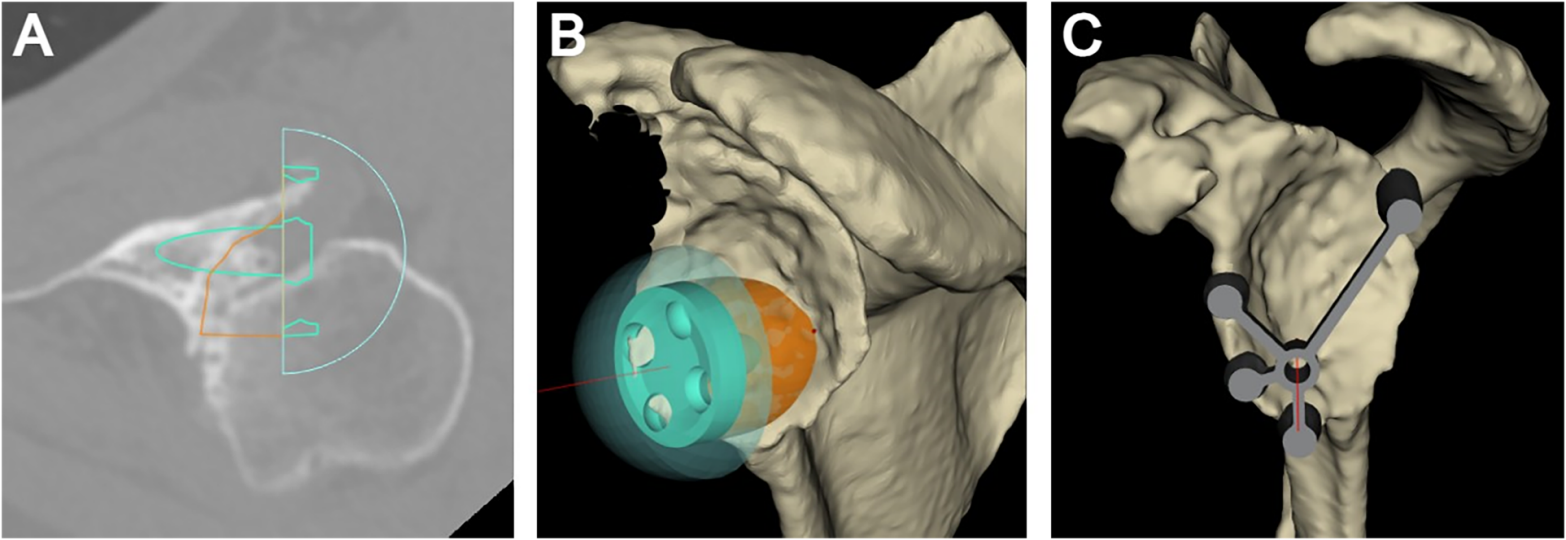
Case example (advanced CTA, left shoulder) outlining the necessary steps taken for preoperative planning. Three-dimensional (3D) planning software reveals 38° of glenoid retroversion and 5° of inferior inclination. (A) The planning software is used to determine the optimal position of the glenoid baseplate in the axillary (ie, the primary plane of deformity in this case) and coronal planes. (B) 3D rendering that includes the patient-specific bone graft (ie, digital graft) with the “point of maximal glenoid defect” denoted by the red dot. (C) The planned location and orientation of the glenoid guide pin are templated. Note is made of the substantial glenoid retroversion.

A high-resolution computed tomography (CT) scan of the operative shoulder is obtained to assess glenoid version and inclination (ie, glenoid deformity). Images are exported in Digital Imaging and Communications in Medicine (DICOM) format into preoperative 3D planning software (Blueprint™, Stryker, Bloomington, MN) which permits accurate and reproducible measurements of version and inclination and the graft dimensions required to address the glenoid deformity.^6,7^ The virtual model is then used to template the planned location and orientation of the glenoid baseplate (Figure 1A). The baseplate position is typically kept within 10° of neutral ante/retroversion, and the central post is positioned parallel to the floor of the supraspinatus fossa (ie, 0° of inclination or slight inferior inclination). A screenshot is saved of the “heat map” which represents the reamer’s contact with the glenoid when positioning the glenoid baseplate within the planning software. This permits the surgeon to preoperatively assess the location, orientation and amount of high-side reaming and can be used as an intraoperative reference during glenoid preparation.^8^ Once the baseplate has been positioned appropriately the “patient-specific bone graft” option is selected and is then evaluated for its shape, angulation, and orientation. The location of the maximum (or deepest) aspect of the bony defect is noted on the plan, which corresponds to the deepest part of the patient-specific graft. A screenshot is taken of this location for future/intraoperative reference (Figure 1B). Once satisfied with the preoperative plan, a patient-specific instrument (PSI) guide is ordered, allowing the surgeon to replicate the planned implant position intraoperatively (Figure 1C). The preoperative plan that has been created is then evaluated as to the final bone graft dimensions, which typically do not include lateralization. As only the height and angle of the bone graft are listed in the planning report, simple trigonometry is applied to determine the width of the triangular bone graft that has been planned.

### Surgical Exposure and Graft Harvest

After regional (ie, interscalene nerve block) and general anesthesia, the patient is placed in the beach chair position with the head of the bed at 20° to 40° of elevation. A standard deltopectoral approach is utilized. The biceps tendon, if present, is either tenotomized or tenodesed to the pectoralis major tendon (ie, surgeon preference). A subscapularis peel is performed, which includes careful capsular release along the humeral neck, to expose the glenohumeral joint. With gentle adduction, extension, and external rotation of the shoulder, the shoulder is dislocated and the humeral head and proximal humerus exposure is optimized. As the arthritic humeral head is often quite sclerotic, an initial humeral cut is made just under the eburnated subchondral bone to expose a flat cancellous bone surface approximately corresponding to the orientation of the anatomic neck. The 29 mm pin guide is then used to mark the location of the central post. Depending on the size of the baseplate to be used, a 29 mm bell saw is used to harvest an appropriately sized dowel (ie, cylindrical shape) of cancellous bone from the humeral head. The graft is then transferred to the bone graft cutting guide for final graft preparation. Humeral preparation follows in the standard fashion and the trial stem is left in place to protect the proximal humerus.

### Graft Preparation and Implantation

**Figure 2.**
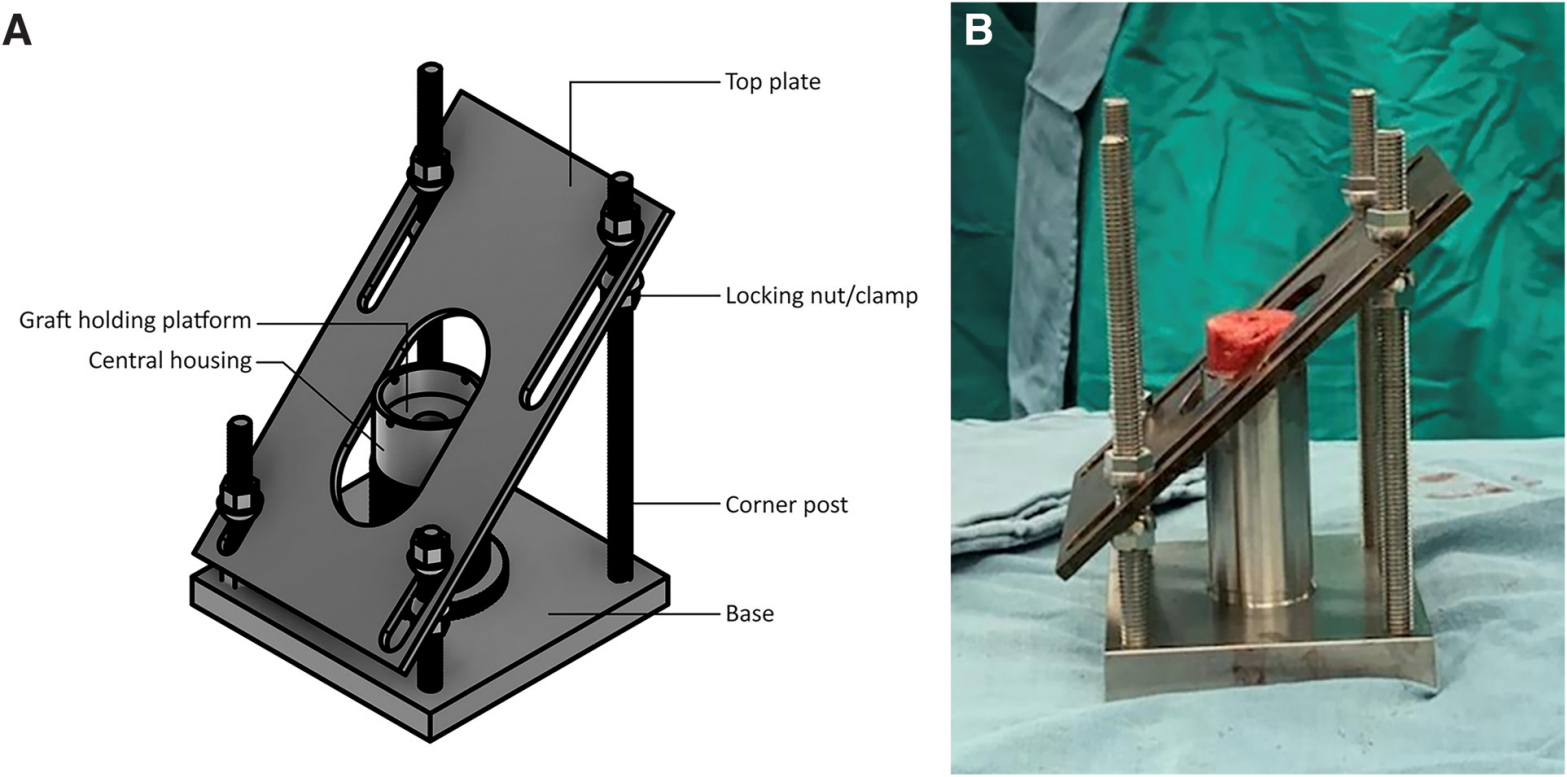
(A) Illustration and (B) intraoperative view of the external bone graft cutting guide (patent pending, BBH technologies Inc.).

**Figure 3.**
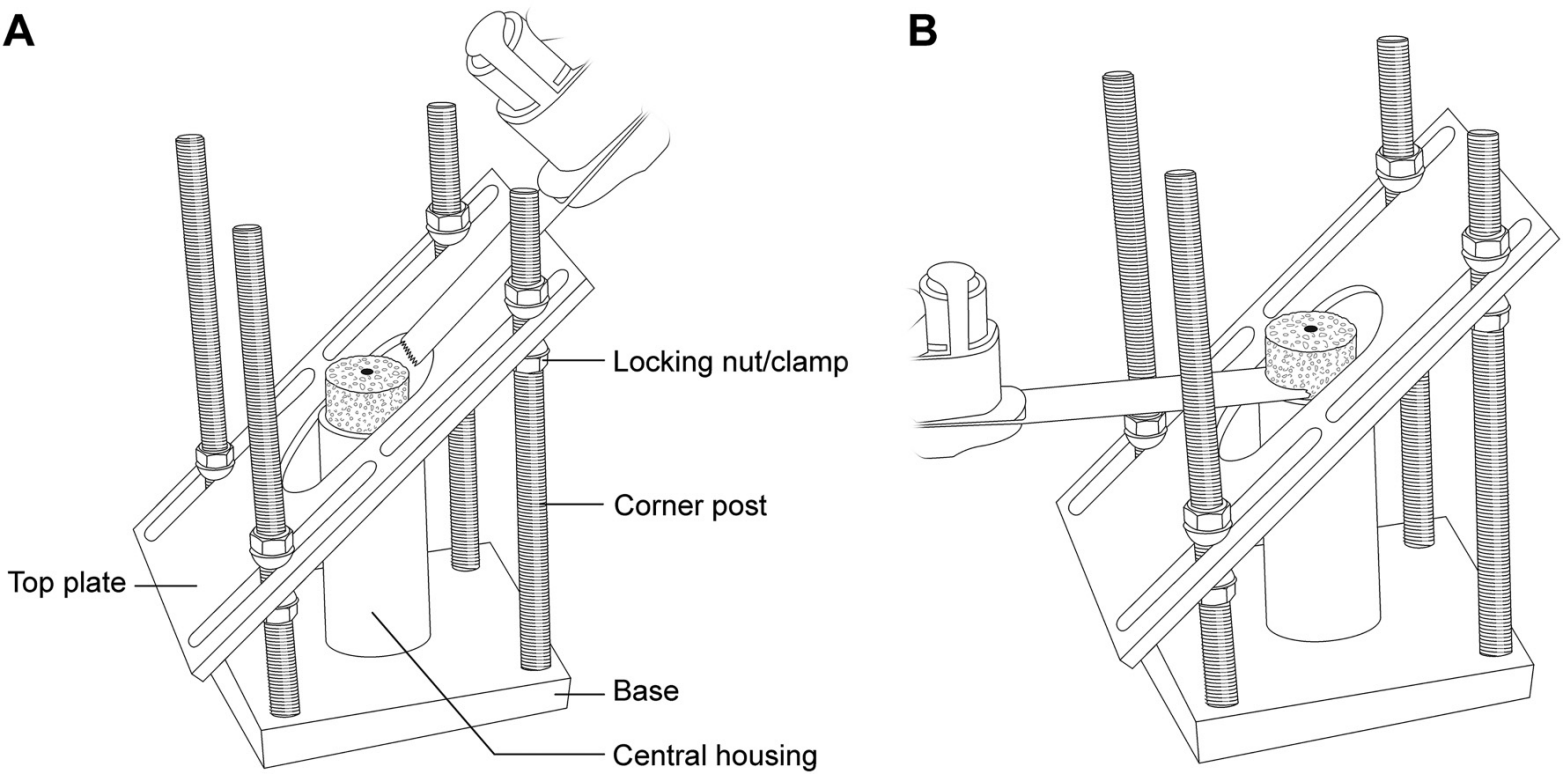
(A) Illustration of bone graft preparation once the graft is secured within the central housing. The first cut is made along the cutting/top plate once the slope of the cut has been set to match the preoperative plan. (B) The second cut is made along the top surface of the central housing to complete a horizontal step-cut if planning to create a biplanar graft.

**Figure 4.**
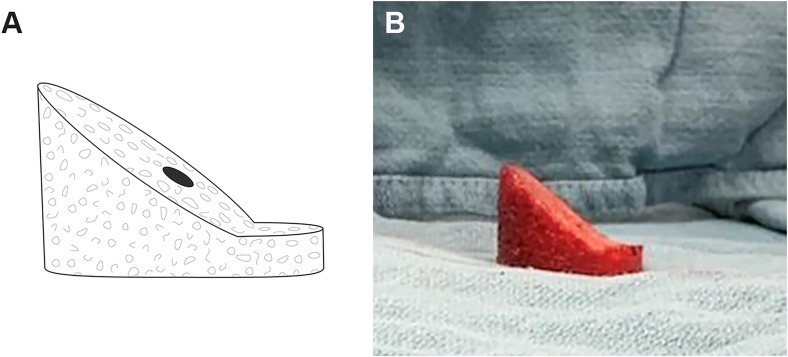
(A) Illustration and (B) intraoperative view of the biplanar graft created to match the preoperative plan presented in Figure 1 to address the substantial glenoid retroversion.

The external bone graft cutting guide (patent pending, BBH Technologies Inc.) is assembled on the back table (Figure 2); this surgical guide was developed to successfully create patient-specific bone grafts according to the preoperative plan. A small fragment screwdriver is used to adjust the graft holding platform to the depth that corresponds to the desired additional lateralization as preoperatively planned (ie, typically 2–3 mm). Graft thickness should not be less than 3–4 mm at its thinnest region to prevent graft fracture and no more than 15–20 mm at the “point of maximal glenoid defect” to prevent overstuffing (ie, glenoid lateralization) of the joint. The locking nuts/clamps are adjusted on each corner post to support the cutting/top plate at the desired height and angulation. The height and orientation of the cutting plate will determine the direction of the graft cuts (Figure 2A). Using a sterile marking pen, the desired minimum and maximum graft thickness is marked on the bone graft, which is then placed on the graft holding platform of the cutting guide (Figure 2B). Using a long saw blade freehand (ie, not attached to power), place it flush against the cutting/top plate surface, bringing it into contact with the graft from multiple angles to ensure the top plate is positioned properly to match the planned graft dimensions to within 1 mm to 2 mm before cutting the graft. If the graft dimensions contacted by the saw blade are not as desired, the cutting plate can be readjusted accordingly. Insert K-wires through the provided holes within the central housing of the guide into the graft to stabilize the graft as needed. When satisfied with the cutting guide position and the planned graft, cut the graft using the saw with the cutting/top plate surface as a guide at the desired angle (Figure 3A). The upper flat surface of the central housing which holds the graft can also be used to cut a horizontal step on the graft (Figure 3B). Utilizing the cutting guide in this fashion (ie, step-cut) will create a biplanar graft (Figure 4). This ‘step-cut’ portion of the graft will then match the eccentrically reamed glenoid surface when the graft is inserted (described below). Note that other graft shapes (ie, trapezoid) can be created using the external cutting guide and are dependent on the glenoid deformity being addressed and surgeon preference.

Following graft preparation, the glenoid is exposed and cleared of labrum and residual cartilage. The bony anatomy of the glenoid including rim osteophytes is preserved to ensure accurate positioning and seating of the PSI guide and placement of the glenoid guide pin (ie, replicating the preoperative plan). Next, the anterior/inferior glenoid is prepared with eccentric reaming consistent with the preoperative plan. If any high-side reaming has been planned, the surgeon should take care at this point to replicate the heat map generated by the plan; otherwise over or underreaming will result in a mismatch of the graft and improper seating. As noted, we typically ream the native glenoid surface to the leading edge of the post of the glenoid baseplate on the high side (ie, the paleoglenoid). The region of new glenohumeral contact (ie, the neoglenoid) that represents the region of glenoid erosion and/or deformity is preserved to allow proper seating of the harvested graft and thereby optimize baseplate stability. Residual cartilage and labrum are removed from the neoglenoid which is then drilled with a 2 mm drill bit to create a bleeding bone surface, which has been demonstrated to aid in bone graft incorporation.^9^ The glenoid surface is then thoroughly assessed and the “point of maximal glenoid defect” is marked with electrocautery, which will be located approximately 90° from the line formed by the eccentric ream.^8^ The graft is then placed over the central post of the glenoid baseplate and oriented to ensure that the thickest portion of the graft will be inserted at the marked “point of maximal glenoid defect.” A long hydroxyapatite (HA)-coated central post is preferred over a central screw in such cases to ensure at least 1 cm of the post gains sufficient purchase into the patient's native glenoid bone at the time of implant insertion. Furthermore, an HA-coated post theoretically permits bony ongrowth over time from both the native glenoid and the bone graft versus a central screw that obtains variable bone purchase into the native glenoid and no bony ongrowth. The baseplate can also be oriented independent of the graft as needed to facilitate screw insertion into the best bone within the glenoid/scapula. The baseplate is then gently impacted until fully seated allowing the cancellous bone graft to compress and contour to any small residual undulations in the posterior/posterosuperior glenoid surface. A standard (ie, centered) glenosphere is then inserted and secured to evenly distribute loads to the underlying bone graft, which will theoretically improve graft incorporation and limit resorption (ie, as per Wolff's law). Lateralized spheres are not typically utilized to avoid excessive glenoid lateralization, especially in cases where 2–3 mm of glenoid lateralization has been built into the plan. A trial reduction is performed prior to insertion of the definitive humeral component ensuring adequate soft-tissue tensioning, stability, and absence of perimeter impingement. The joint and surgical wound is then thoroughly irrigated and closed in layers in the standard fashion.

## Results

### Clinical Case

**Figure 5.**
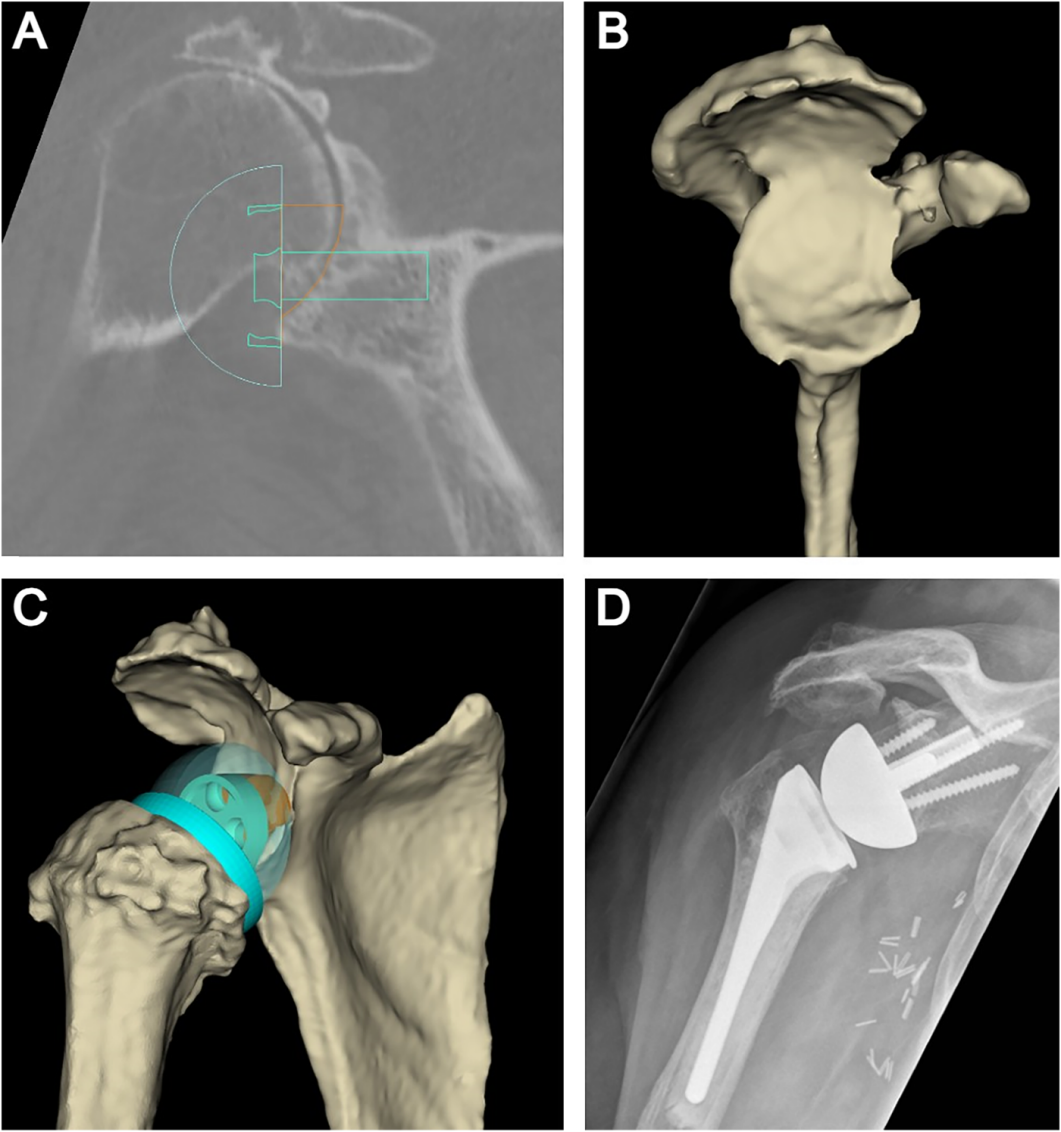
(A) 3D planning software in the coronal plane is used to determine the optimal position of the glenoid baseplate to correct glenoid inclination and avoid superior tilt. (B) 3D rendering with subtraction of the humeral head reveals the primary glenoid deformity in the posterior-superior quadrant (Favard Type E3 glenoid) in this case example. (C) Patient-specific bone graft (ie, digital graft) that is created using the planning software to address the glenoid deformity. (D) Postoperative anteroposterior radiograph of the shoulder revealing the patient-specific bone graft with correction of glenoid inclination.

An interesting case that demonstrates the above-described surgical technique and the wide array of glenoid deformities that can be managed with utilization of the external bone graft cutting guide involves a 79-year-old, right-hand dominant female who presented with right shoulder pain and loss of function resulting from end-stage CTA. Preoperative planning using 3D CT software revealed 21° of superior inclination and 4° of glenoid retroversion (Favard type E3 glenoid). A preoperative plan was developed as per the parameters described above. The patient underwent an RSA with bone grafting utilizing the above technique. Excellent seating of the trapezoid-shaped bone graft and glenoid baseplate was achieved. Peripheral screws were inserted in standard fashion and a 39 mm nonlateralized (ie, centered) glenosphere was then secured onto the baseplate which ensured joint stability without perimeter impingement. There were no intraoperative or postoperative complications encountered. At the latest follow-up, the patient's pain was well-controlled and was progressing well with the postoperative rehabilitation program. Preoperative planning software images and postoperative radiographs are demonstrated in Figure 5.

The senior authors have developed and used this surgical technique with the described external guide over the past 2 years in patients with complex glenoid deformities in the setting of RSA. In cases where this guide has been used, patients have been enrolled in a prospective clinical cohort study. As this clinical study is ongoing and data collection incomplete, the results have not been finalized and/or published. However, to date, the authors have not observed complications or adverse events directly related to the bone graft (ie, fracture, nonunion or substantial resorption) and/or the overlying glenoid baseplate (ie, baseplate failure), nor has there been any other complications noted in this study cohort. In the event of an intraoperative graft fracture that compromises baseplate support, one must consider converting to a metal-augmented glenoid baseplate to ensure the goals of surgery (ie, a stable construct without perimeter impingement) have been achieved at the end of the procedure. If a metal-augmented baseplate is not available, allograft bone (eg, humeral head allograft) can also be utilized in the same manner as an autograft to address the glenoid deformity.

## Discussion

In reverse shoulder arthroplasty, glenoid component position is vital to improved implant longevity.^10–12^ Avoiding superior tilt and perimeter impingement are key technical recommendations during implant insertion.^13–16^ Walch et al^6^ have shown that 3D planning, and the use of patient-specific guides improve glenoid component position, especially in cases with substantial glenoid deformity.

When confronted with substantial glenoid bone loss in patients undergoing RSA, the surgeon must decide how to reconstruct the defect using either a metal augment or bone graft. Metal augmentation can be accomplished with a standard (ie, preset size/angulation) wedged or patient-matched implant. Metal augmentation with a wedged implant not only limits the surgeon's selection of implant size and shape that have been standardized by the manufacturer but also requires glenoid preparation for adequate implant seating.^17,18^ Patient-matched metal implants are also now available for complex primary and revision procedures,^19^ which are intended to preserve glenoid bone during reconstruction as the implant is designed to seat perfectly on the deformed glenoid surface with little to no bone preparation. This offers the additional advantage of decreased surgical time and may be less technically challenging than bone grafting. Unfortunately, the cost of patient-matched components can be prohibitive, and there are few clinical outcome studies available to support this novel technology and justify its cost.

The use of bone graft in primary arthroplasty cases to address glenoid deformity commonly involves harvesting autograft from the patient's humeral head followed by contouring the graft to match the glenoid defect. The goals of this procedure are to correct the glenoid deformity and to restore glenoid bone stock.^20^ The use of autograft is more economical compared to a metal augment and has been proven clinically effective in the peer-reviewed literature.^21^ The disadvantage of using a bone graft (ie, autograft or allograft) is that it is both technically demanding and time-consuming. This is partially due to the absence of a reproducible technique for the creation of a patient-specific graft. Furthermore, even though rates of bone graft incorporation are high,^22,23^ there is still some concern regarding graft resorption.^24,25^ Boileau et al^26^ described a technique for the use of angled bony-increased offset RSA (angled BIO-RSA) to address multiplanar glenoid deformity. The authors demonstrated that the use of an angled bone graft (ie, trapezoid shape) resulted in a reliable and predictable union of the graft with the native glenoid, correction of malalignment, and successful lateralization of the glenoid component with low complication rates.

In a recent 2023 systematic review, the clinical results and complications were compared between patients undergoing RSA with bone grafting (*n* = 401) and augmented baseplates (*n* = 251).^27^ All 19 studies included in this review were case series (Level IV evidence) with an average follow-up of 23.1 months (bone graft group) and 29.5 months (augmented group). Despite differences in the overall glenoid deformity types addressed in each treatment group, the overall complication (11.7% vs 11.8%) and revision (4.5% vs 3.7%) rates were equivalent for the bone graft and augmented baseplate groups, respectively. Bone graft nonunion and failure to incorporate occurred in 5.5% of cases; however, bone graft incorporation did not correlate with component (eg, baseplate) failure. Both groups also demonstrated similar improvements in their overall clinical outcomes (ie, range of motion and patient-reported outcome scores).

In 2022, Van De Kleut et al^28^ reported on the results of a randomized controlled trial (Level I evidence) that failed to demonstrate any substantial differences in clinical outcomes between metal-augmented baseplates and BIO-RSA at 2 years follow-up. All bone grafts demonstrated structural integrity without evidence of glenoid lucency and neither treatment group had evidence of baseplate failure at the latest (2-year) follow-up. Due to its low cost, nearly universal availability, and excellent clinical outcomes, we prefer to use bone grafts in these challenging primary RSA cases with large glenoid defects. In addition, the use of bone graft can also be used to restore glenoid bone stock in the revision setting but with less reliable results.^29^ Previous bone grafting techniques have been described in the literature to reconstruct severe glenoid bone defects; however, such techniques are not patient-specific and rely on imprecise “free-hand” methods. The technique described herein allows the surgeon to reliably create the preoperatively planned patient-specific bone graft with the desired amount of lateralization of the baseplate.

Three-dimensional CT-based software exists for preoperative planning of RSA and has demonstrated its utility through its widespread use.^30^ Using Blueprint™ (Stryker, Bloomington, MN) planning software, there is an option to select a “patient-specific bone graft” (ie, digital graft) without a standardized, reproducible, or precise way to physically create the graft intraoperatively. In the present study, we have introduced a novel surgical technique that will allow the surgeon to create a bone graft that accurately matches nearly any glenoid defect that is encountered. To date, we have demonstrated excellent bony reconstruction of the glenoid in a prospective cohort of patients, with excellent short-term clinical and radiographic follow-up; as patient recruitment is still ongoing, the results of this study have not been finalized and/or published.

The described surgical technique that utilizes an external guide to prepare a patient-specific bone graft offers advantages over current (ie, “free-hand”) techniques. First, the surgeon can make the bone graft of the exact size and shape of their choice to manage the defect encountered (ie, an “individualized” approach to the glenoid deformity) and precisely match the computer-generated plan (ie, “digital graft”). This provides a reproducible method to address glenoid deformities and improve implant seating and stability. Second, the graft's shape can be adjusted, if necessary (ie, different from the preoperative plan), by way of the modularity of the external guide. For example, the amount of glenoid lateralization can be adjusted from the computer-derived bone graft simply by raising/lowering the graft holding platform within the central housing of the guide (Figure 2). The disadvantage of this technique includes its dependency on an external guide/reusable device that is subject to all the expected concerns related to surgical equipment (eg, wear and breakage with repeated use, storage space requirements, and costs). Another important disadvantage for surgeons to consider is the inherent learning curve involved with any new surgical technique and/or the use of new surgical equipment.

## Conclusion

In this article, we present a novel surgical technique utilizing an external guide that can be used to successfully create patient-specific bone grafts for reverse shoulder arthroplasty, particularly in cases with severe glenoid bone loss. With the use of this adjustable and reusable guide, addressing glenoid bone loss with either autograft or allograft presents the shoulder surgeon with a safe, reliable, and simple alternative to metal augmentation in these challenging primary or revision cases. Future work includes the refinement of existing preoperative planning software to include automatic computation of bone graft dimensions and subsequently the required adjustments of the guide's cutting plate to further improve efficiency and accuracy of graft preparation intraoperatively.
